# Alcohol-Screening Instruments for Pregnant Women

**Published:** 2001

**Authors:** Grace Chang

**Affiliations:** Grace Chang, M.D., M.P.H., is an associate professor of psychiatry at Harvard Medical School, Department of Psychiatry, Brigham and Women’s Hospital, Boston, Massachusetts

**Keywords:** prenatal alcohol exposure, prenatal diagnosis, alcohol use test, identification and screening for AOD (alcohol or other drug) use, specificity and sensitivity of measurement, breath alcohol analysis, AODR (alcohol- or other drug-related) biological markers

## Abstract

According to new studies, even low levels of prenatal alcohol exposure can negatively affect the developing fetus, thereby increasing the importance of identifying women who drink during pregnancy. In response, researchers have developed several simple alcohol-screening instruments for use with pregnant women. These instruments, which can be administered quickly and easily, have been evaluated and found to be effective. Because of the potential adverse consequences of prenatal alcohol exposure, short screening questionnaires are worthwhile preventive measures when combined with appropriate followup.

Screening pregnant women for alcohol use has become of increasing importance, because new research indicates that even low levels of prenatal alcohol exposure can negatively affect the developing fetus. Adverse effects of prenatal alcohol exposure can range from subtle developmental problems, or fetal alcohol effects, to full-blown fetal alcohol syndrome. In addition, scientists and clinicians have found that certain neurobehavioral outcomes associated with prenatal alcohol exposure can persist in the affected person into adolescence (Sampson et al. 1994) and adulthood (Kelly et al. 2000).

Because no universally safe level of alcohol consumption during pregnancy has been identified (Stratton et al. 1996), the U.S. Surgeon General and the Secretary of Health and Human Services recommend abstinence both before conception and throughout pregnancy (Stratton et al. 1996; Ebrahim et al. 1998). However, approximately 20 percent of women drink some alcohol during pregnancy, and the rate of frequent drinking (i.e., seven or more drinks per week or five or more drinks per occasion) by pregnant women has increased substantially, from 0.8 percent in 1991 to 3.5 percent in 1995 (Ebrahim et al. 1998; Centers for Disease Control and Prevention 1997). This rise in the rate of alcohol consumption among pregnant women coincides with growing evidence of the negative effects of low-to-moderate alcohol consumption during pregnancy.

Increasingly sophisticated research has improved scientific and clinical understanding of the adverse consequences of prenatal alcohol exposure. The term “pregnancy risk drinking” (i.e., drinking during pregnancy at levels considered risky to the fetus) was previously defined as the consumption of 1 ounce or more of alcohol (i.e., two or more drinks) per day (Sokol et al. 1989), but more recent findings show that even lower levels of alcohol consumption can lead to negative pregnancy outcomes (Charness et al. 1994; Wong et al. 1995; Ikonomidou et al. 2000; Jacobson and Jacobson 1994). A study of more than 5,000 pregnant women who consumed alcohol moderately (defined as at least 3.5 drinks per week) demonstrated that the women who drank more than 3.0 drinks per week increased significantly their risk of first-trimester spontaneous abortion (Windham et al. 1997).

Identifying women who drink at risky levels during pregnancy poses special challenges, however, particularly because the definition of pregnancy risk drinking has been refined over time. In addition, screening for *any* alcohol use during pregnancy is difficult. This article discusses the difficulties involved in screening pregnant women for alcohol use; details some of the questionnaires, or instruments, available to facilitate alcohol screening in this population; and briefly describes a few laboratory tests used for detecting alcohol use among pregnant women.

## Complications of Screening Pregnant Women for Alcohol Use

A key complication in screening pregnant women for alcohol use arises from the fact that the traditional alcohol-screening questionnaires—such as the Michigan Alcoholism Screening Test (MAST) (Selzer 1971) and the CAGE^1^ (Ewing 1984)—are less effective in identifying drinking problems among women than among men. This discrepancy is attributable to the fact that these instruments were developed among men, who have different patterns of alcohol consumption and different thresholds for problem drinking than women (Babor et al. 1989). In addition, these instruments were developed to detect alcohol dependence, which is relatively uncommon among pregnant women (Ebrahim et al. 1998). Because of biological differences between women and men, the same quantity of alcohol consumed over the same time period produces higher blood alcohol levels in women than in men (Graham et al. 1998). Women are also more sensitive than men to alcohol-related organ damage, such as cardiomyopathy and myopathy (Urbano-Marquez et al. 1995; Hanna et al. 1992). Therefore, alcohol-screening-instrument cutoff scores (i.e., the values that clinicians use to define a positive result from a screening instrument) most likely need to be set differently for men and women and particularly for pregnant women (Bradley et al. 1998).

A second complication faced by researchers is that many women alter their alcohol consumption once they learn that they are pregnant. Consequently, inquiries about drinking patterns before pregnancy confirmation are potentially more accurate measures of first-trimester drinking (Day et al. 1993). Women are also likely to deny or minimize their drinking during pregnancy out of embarrassment (Morrow-Tlucak et al. 1989). Even moderate drinkers may underreport alcohol consumption during pregnancy (Verkerk 1992). Data from a sample of 361 mothers suggest that women who report drinking more than 1.3 drinks per week during pregnancy actually may be drinking at levels high enough to incur risk for alcohol-related birth defects (Jacobson et al. 1991). For example, 53 percent of the women who reported drinking more than 1.3 drinks per week during pregnancy reported higher levels of consumption when interviewed retrospectively.

A third complication is that standard questions about quantity and frequency of alcohol consumption are unlikely to be helpful when screening pregnant women for alcohol use. The widely used American College of Obstetricians and Gynecologists (ACOG) Antepartum Record poses three questions about alcohol use: (1) the amount of alcohol consumed per day before pregnancy, (2) the amount of alcohol consumed per day during pregnancy, and (3) the number of years of alcohol use. The Antepartum Record has a fill-in-the-boxes format designed to gather standard clinical information on obstetric patients. However, compared with the 13-item Prenatal Alcohol Use Interview, the ACOG Antepartum Record is less successful in identifying prenatal alcohol use. Researchers suggest that the difference in findings between the two instruments may be attributable to the format of the ACOG Antepartum Record and its lack of guiding questions: the ACOG instrument requires a skilled interviewer in order to elicit accurate responses about drinking during pregnancy (Budd et al. 2000).

A final complication is that obstetricians inconsistently screen their patients for alcohol use during pregnancy. One goal of Healthy People 2000 was to increase obstetricians’ rate of screening for alcohol use to 75 percent, from the 1987 rate of 34 percent (Stratton et al. 1996). Progress toward this goal has not yet been reported.

In response to the need for increased alcohol screening among pregnant women, researchers have developed several alcohol-screening instruments specifically for use with this population.

## Screening Instruments

The screening instruments described in this section were tested in diverse clinical populations and may help identify women using alcohol during pregnancy. These instruments vary in that they were designed to detect different levels of alcohol use and, therefore, differ in how they define pregnancy risk drinking.

In general, a positive screen does not indicate an alcoholism diagnosis; rather, it may signal to a physician or other health care practitioner the need to discuss pregnancy risk drinking with a patient. Routine use of screening questionnaires in clinical practices may reduce the stigmatization of asking patients about alcohol use and result in more accurate and consistent evaluation.

Sensitivity and specificity are two important properties of every screening instrument. The *sensitivity* of a screening test refers to the probability that a person who should test positive, does so (i.e., the sensitivity of a screen for pregnancy risk drinking is the probability that a woman who is a risk drinker tests positive). The *specificity* of a screening test is the probability that a person who should test negative, does so (i.e., the probability that a woman who is not a risk drinker tests negative) (Rosner 1990).

### The T-ACE

The T-ACE was the first validated sensitive screen for risk drinking (defined as alcohol consumption of 1 ounce or more per day) developed for use in obstetric-gynecologic practices (Sokol et al. 1989). An obstetrician developed the T-ACE after observing that asking patients about their tolerance to the intoxicating effects of alcohol did not trigger denial. The “socially correct” answer is not known (patients do not feel stigmatized to answer honestly), and tolerance reflects a pattern of drinking.

The four T-ACE questions (see T-ACE textbox) take less than 1 minute to ask. The T-ACE is positive with a score of 2 or more points. One point is given for each affirmative answer to the A, C, or E questions. Two points are given when a pregnant woman reports that more than two drinks are necessary for her to feel “high” or experience the intoxicating effects of alcohol.

**T-ACE**

**Table:** 

**T**	**Tolerance:** How many drinks does it take to make you feel high?
**A**	Have people **Annoyed** you by criticizing your drinking?
**C**	Have you ever felt you ought to **Cut down** on your drinking?
**E**	**Eye opener:** Have you ever had a drink first thing in the morning to steady your nerves or get rid of a hangover?

The T-ACE is used to screen for pregnancy risk drinking, defined here as the consumption of 1 ounce or more of alcohol per day while pregnant. Scores are calculated as follows: a reply of “More than two drinks” to question T is considered a positive response and scores 2 points, and an affirmative answer to question A, C, or E scores 1 point, respectively. A total score of 2 or more points on the T-ACE indicates a positive outcome for pregnancy risk drinking.

SOURCE: Sokol et al. 1989.

Researchers initially evaluated the T-ACE in a sample of 971 African-American women attending an inner-city antenatal clinic. The researchers administered both the MAST and CAGE as well as asked the T-ACE tolerance question, “How many drinks does it take to make you feel high?” The T-ACE was not administered as an independent instrument; instead, both the sensitivity and specificity of the T-ACE were calculated from the subjects’ responses to the tolerance question as well as to the annoyed, cut-down, and eye-opener questions from the CAGE questionnaire. The T-ACE proved to be superior to both the MAST and CAGE in identifying pregnancy risk drinking (i.e., defined as alcohol consumption of more than 1 ounce daily). Table 1 summarizes the study’s findings.

We subsequently tested the T-ACE as a self-administered, independent screening tool embedded in a health-habits survey with questions about smoking, stress, weight, and dietary habits in a more socially and ethnically diverse obstetric population—350 women initiating prenatal care at the Brigham and Women’s Hospital in Boston, Massachusetts (Chang et al. 1998).

We compared the sensitivity and specificity of the T-ACE with the sensitivity and specificity of three other popular methods of screening for alcohol use in other clinical settings: (1) the Alcohol Use Disorders Identification Test (AUDIT) (Babor et al. 1992), (2) the Short Michigan Alcoholism Screening Test (SMAST) (Selzer et al. 1975), and (3) a review of the patient’s medical record. Researchers gave each participant the AUDIT and SMAST independently as well as reviewed the participant’s medical record. The three criteria used to evaluate the T-ACE, AUDIT, SMAST, and medical record were as follows: (1) alcohol abuse or dependence diagnoses as defined according to the *Diagnostic and Statistical Manual of Mental Disorders, Third Edition, Revised* (DSM-III-R) (American Psychiatric Association 1987), which the subject could meet at any point in her lifetime; (2) risk drinking, defined as having more than two drinks per drinking day before pregnancy; and (3) current drinking (i.e., any alcohol consumption during pregnancy).

Table 2 summarizes the sensitivity and specificity of the T-ACE, AUDIT, SMAST, and medical record for the three criteria. In addition, sensitivity and specificity for varying cut-off scores for the T-ACE and AUDIT are listed (e.g., in response to the tolerance question in the T-ACE, “more than 2 drinks” would be a positive response in one scoring method and “2 or more drinks” would be a positive response when using a different scoring method). With “tolerance” defined as “2 or more drinks to feel intoxicated,” the T-ACE was the most sensitive instrument to detect current alcohol consumption, risk drinking, and lifetime DSM-III-R alcohol diagnoses. However, it was also the least specific.

The ideal screening test would be both highly sensitive and highly specific; however, any given test usually has a trade off. Screeners typically give priority to sensitivity if it is important to identify a condition, even if more false positives are subsequently identified. However, if insufficient resources are available to evaluate all patients who screen positive, then specificity may be considered more important (Russell 1994). Thus, the T-ACE, with a positive response to the tolerance question defined as “more than 2 drinks,” offers the best balance of sensitivity and specificity.

The T-ACE is a valuable and efficient tool for identifying alcohol use among pregnant women; in addition, it demonstrates acceptability and accuracy in identifying a range of alcohol-use levels in diverse obstetric populations. The questions are easy to both remember and score and can be asked by an obstetrician or nurse in 1 minute. Women waiting for their prenatal appointments, for example, could be asked to complete the T-ACE as part of a routine patient questionnaire to be reviewed during the visit.

### The TWEAK

The TWEAK is a five-item screening tool that includes questions from the MAST, CAGE, and T-ACE (see TWEAK textbox). The TWEAK is designed to detect alcoholism or heavy drinking and was first tested in three male and female samples randomly selected from three groups: (1) alcoholics in treatment at a county medical center; (2) patients at two primary health care centers; and (3) the general population of the Buffalo, New York, metropolitan area (Chan et al. 1993). Subsequent evaluation of the TWEAK has revealed its promise as a screening tool for identifying pregnant women who are at-risk drinkers, defined as those consuming 1 ounce of alcohol or more daily (Russell et al. 1994).

**TWEAK**

**Table:** 

**T**	**Tolerance:** How many drinks can you hold?
**W**	Have close friends or relatives **Worried** or complained about your drinking in the past year?
**E**	**Eye Opener:** Do you sometimes take a drink in the morning when you get up?
**A**	**Amnesia:** Has a friend or family member ever told you about things you said or did while you were drinking that you could not remember?
**K(C)**	Do you sometimes feel the need to **Cut down** on your drinking?

The TWEAK is used to screen for pregnancy risk drinking, defined here as the consumption of 1 ounce or more of alcohol per day while pregnant. Scores are calculated as follows: A positive response to question T on Tolerance (i.e., consumption of more than five drinks) or question W on Worry yields 2 points each; an affirmative reply to question E, A, or K scores 1 point each. A total score of 2 or more points on the TWEAK indicates a positive outcome for pregnancy risk drinking.

SOURCE: Chan et al. 1993.

The TWEAK is scored on a 7-point scale. On the tolerance question, 2 points are given if a woman reports that she can consume more than five drinks without falling asleep or passing out. A positive response to the worry question yields 2 points, and positive responses to the last three questions yield 1 point each. A woman who has a total score of 2 or more points is likely to be an at-risk drinker.

Like the T-ACE, the TWEAK asks about tolerance to the effects of alcohol. In one study of 4,743 African-American women of low socioeconomic status who were given the MAST, the CAGE, and the T-ACE tolerance question, the calculated sensitivity and specificity of the TWEAK were 79 percent and 83 percent, respectively, in contrast to the calculated 70-percent sensitivity and 85-percent specificity of the T-ACE. Periconceptional risk drinking, defined as 1 ounce or more of alcohol consumption per day or 14 drinks per week during a typical week before pregnancy (Russell et al. 1994), was the criterion standard (i.e., this was the level of drinking that the instruments were trying to detect). The ability to generalize these findings is limited. This is attributable to the homogenous makeup of the sample, the fact that neither the T-ACE nor the TWEAK were administered as independent instruments, and the definition of periconceptional risk drinking, which other researchers have subsequently updated to 0.5 ounces of alcohol per day (Hankin and Sokol 1995).

The TWEAK does not appear to offer any significant advantages over the T-ACE. Most studies investigating the TWEAK’s performance have relied on a definition of risk drinking that does not reflect more current research. Nonetheless, it offers another option for clinicians.

## Other Screening Questionnaires

Research has not established the utility of other screening questionnaires—the CAGE, SMAST, AUDIT, and Prenatal Alcohol Use Interview—for pregnant women. The CAGE and the SMAST are popular self-report measures of alcoholism and are well studied in alcoholic and nonalcoholic subjects and among males (Bradley et al. 1998). The AUDIT is a 10-item questionnaire that identifies harmful and hazardous drinking during the past year and has been validated in six countries (Cherpitel 1995). The Prenatal Alcohol Use Interview is a 13-item questionnaire that has been tested in a sample of 56 women thus far and requires further evaluation (Budd et al. 2000).

Two large studies of disadvantaged, minority, obstetric patients (Hankin and Sokol 1995; Russell et al. 1996) reported that the calculated sensitivity and specificity of the T-ACE and TWEAK were superior to the CAGE in identifying risk drinking (defined as 1 ounce or more of alcohol consumption per day). In another study, we gave the SMAST, AUDIT, and T-ACE questions independently to 350 pregnant women (Chang et al. 1998) and calculated how well each of the three instruments could predict lifetime DSM-III-R alcohol diagnoses and any drinking during pregnancy. The SMAST did not perform better than chance as a predictor for either of the two drinking categories. Although the AUDIT had good predictive ability, the definition of a “positive” score on the AUDIT for drinking pregnant women remains to be identified and confirmed through further research.

## Laboratory Tests for Detecting Alcohol Use

Although the central focus of this article is on screening questionnaires, other methods of detecting alcohol use during pregnancy deserve some comment. Use of breath analysis or urinalysis in pregnant patients is not likely to be feasible or acceptable, given the rapid metabolism of alcohol and the pattern of drinking by most pregnant women (i.e., it is unlikely that pregnant women will consume alcohol right before their obstetric appointment) (Testa and Reifman 1996; Lundberg et al. 1997; Strano-Rossi 1999). However, recent research has demonstrated the potential value of maternal blood markers for detecting levels of alcohol use during pregnancy that may result in overt alcohol-related deficits in newborns. However, the most significant and most common result of prenatal alcohol exposure, neurobehavioral dysfunction, is not an outcome recognized in the newborn period. Therefore, research has yet to establish the relevance of these blood markers to the more common fetal alcohol effects (Jones and Chambers 1998; Stoller et al. 1998). (See the article by Bearer on pp. xx-xx of this issue for more information on potential biomarkers to detect alcohol use during pregnancy.)

## Summary

Simple screening questionnaires, such as the T-ACE, provide valuable tools for identifying women who are using alcohol during pregnancy. The T-ACE has been shown to identify any alcohol consumption during pregnancy as well as higher amounts of drinking. Research has demonstrated that any alcohol consumption during pregnancy increases the risk of continued drinking during pregnancy (Chang et al. 1999).

The T-ACE is administered easily. A clinician may either ask the T-ACE questions directly or request that the patient complete the questionnaire while waiting for her appointment. The T-ACE has been tested and demonstrated to be acceptable and effective in both formats.

A positive screen is not an indictment. Rather, it is an opportunity for the clinician and patient to discuss prenatal alcohol exposure. The discussion may lead the clinician to refer the patient for a diagnostic assessment. Or the clinician may offer a brief intervention if the patient does not have a severe alcohol problem. Because most pregnant women are highly motivated to change their behaviors (Hankin et al. 2000), brief interventions (i.e., short counseling sessions) may be especially effective in this population. Given the potential adverse consequences of prenatal alcohol exposure, short screening questionnaires are worthwhile preventive measures.

## Figures and Tables

**Table 1:** Comparison of the T-ACE, CAGE, and MAST in Identifying Pregnancy Risk Drinking

Instrument	Screening for Pregnancy Risk Drinking*		Specificity (%)

	Positive Test Score (points accrued)	Sensitivity (%)	
T-ACE	(> 2)	69	89
CAGE	(> 2)	38	92
MAST	(> 5)	36	96

MAST = Michigan Alcoholism Screening Test.

* Pregnancy risk drinking is defined as the consumption of 1 ounce or more of alcohol per day during pregnancy.

NOTE: The *sensitivity* of a screening test is the probability that a person who should test positive, does so (i.e., the sensitivity of a screen for pregnancy risk drinking is the probability that a woman who is a risk drinker tests positive). The *specificity* of a screening test is the probability that a person who should test negative, does so (i.e., the probability that a woman who is not a risk drinker tests negative) (Rosner 1990).

SOURCE: Sokol et al. 1989.

**Table 2:** Sensitivity and Specificity of the T-ACE, AUDIT, SMAST, and Medical Record

Criterion Standard	Instrument	Sensitivity* (%)	Specificity** (%)
DSM-III-R lifetime alcohol diagnosis	T-ACE (tolerance > 2)	87.8	36.6
	T-ACE (tolerance > 2)	60.0	66.4
	AUDIT (> 11)	7.0	99.6
	AUDIT (> 10)	11.0	99.0
	AUDIT (> 8)	22.6	97.4
	SMAST	14.8	97.9
	Medical record	15.6	93.6
Risk drinking (two drinks per day before pregnancy)	T-ACE (tolerance > 2)	92.4	37.6
	T-ACE (tolerance > 2)	74.3	71.4
	SMAST	11.4	95.9
	Medical record	6.7	89.4
Current alcohol consumption (while pregnant)	T-ACE (tolerance > 2)	89.2	37.8
	T-ACE (tolerance > 2)	60.0	66.9
	AUDIT (> 11)	3.3	97.8
	AUDIT (> 10)	6.7	96.9
	AUDIT (> 8)	15.0	93.9
	SMAST	7.5	94.3
	Medical record	20.0	96.1

SMAST = Short Michigan Alcoholism Screening Test.

* Sensitivity is the probability that a person who should test positive, does so (Rosner 1990).

** Specificity is the probability that a person who should test negative, does so (Rosner 1990).

NOTE: The sensitivity and specificity for varying cutoff scores for the T-ACE and AUDIT are listed (e.g., in response to the tolerance question in the T-ACE, “more than two drinks” would be a positive response in one scoring method and “two or more drinks” would be a positive response under a different scoring method). With tolerance defined as two or more drinks to feel intoxicated, the T-ACE was the most sensitive instrument to detect current alcohol consumption, risk drinking, and lifetime DSM-III-R alcohol diagnoses. However, it was also the least specific.

SOURCE: Chang et al. 1998.

## Footnotes

1 The CAGE screening instrument (Ewing 1984) consists of four questions: (1) Have you ever felt you should Cut down on your drinking?, (2) Have people Annoyed you by criticizing your drinking?, (3) Have you ever felt bad or Guilty about your drinking?, and (4) Have you ever had a drink first thing in the morning to steady your nerves or to get rid of a hangover (Eye opener)?
